# Emergence of zoonotic arboviruses by animal trade and migration

**DOI:** 10.1186/1756-3305-3-35

**Published:** 2010-04-08

**Authors:** Martin Pfeffer, Gerhard Dobler

**Affiliations:** 1 Institute of Animal Hygiene & Veterinary Public Health, University of Leipzig, An den Tierkliniken 1, 04103 Leipzig, Germany; 2 Bundeswehr Institute of Microbiology, Neuherbergstrasse 11, 80937 Munich, Germany

## Abstract

Arboviruses are transmitted in nature exclusively or to a major extend by arthropods. They belong to the most important viruses invading new areas in the world and their occurrence is strongly influenced by climatic changes due to the life cycle of the transmitting vectors. Several arboviruses have emerged in new regions of the world during the last years, like West Nile virus (WNV) in the Americas, Usutu virus (USUV) in Central Europe, or Rift Valley fever virus (RVFV) in the Arabian Peninsula. In most instances the ways of introduction of arboviruses into new regions are not known. Infections acquired during stays in the tropics and subtropics are diagnosed with increasing frequency in travellers returning from tropical countries, but interestingly no attention is paid on accompanying pet animals or the hematophagous ectoparasites that may still be attached to them. Here we outline the known ecology of the mosquito-borne equine encephalitis viruses (WEEV, EEEV, and VEEV), WNV, USUV, RVFV, and Japanese Encephalitis virus, as well as Tick-Borne Encephalitis virus and its North American counterpart Powassan virus, and will discuss the most likely mode that these viruses could expand their respective geographical range. All these viruses have a different epidemiology as different vector species, reservoir hosts and virus types have adapted to promiscuous and robust or rather very fine-balanced transmission cycles. Consequently, these viruses will behave differently with regard to the requirements needed to establish new endemic foci outside their original geographical ranges. Hence, emphasis is given on animal trade and suitable ecologic conditions, including competent vectors and vertebrate hosts.

## Background

During the last decades the appearance of new infectious diseases and an increasing invasion of diseases into new areas created a new category of pathogens: emerging and re-emerging pathogens. Most of the emerging viruses are zoonotic which means they can infect both animals and humans [1]. As outlined in detail in the examples provided below, humans are dead-end hosts in most cases. Hence, in the case of emerging viruses, zoonotic is mainly defined as transmission of viruses from animals to humans rather than vice versa [2]. Among emerging viruses, arboviruses play a major role. Arboviruses are defined as viruses that survive in nature by transmission from infected to susceptible hosts (vertebrates) by certain species of arthropods (mosquitoes, ticks, sandflies, midges etc.). The viruses multiply within the tissues of the arthropod to produce high titres of virus in the salivary glands and are then passed on to vertebrates (humans and animals) by the bites of the arthropods [3].

To establish and maintain an arbovirus transmission cycle three factors are essential: the arbovirus, the arthropod, and the vertebrate. Usually, these three components have a rather complex relationship including factors such as the vector competence for the particular virus and the susceptibility of the vertebrate host for the virus (producing a high-level viremia to allow other vectors to become infected). As prerequisite for continuous circulation of the virus between arthropod vector and vertebrate host, all factors must be available in sufficient numbers, at the same time and at the same place. Scientifically speaking, a formula describing the vector capacity has to yield high positive values to lead to reproduction rates above 1 for the particular arbovirus [4-7]. Taking all this together, the chance for such a scenario, i.e. the establishment of a new endemic transmission cycle, are very low in general and reports about a new "intruder" are rare. However, the recent introduction of e.g. West Nile virus into the Americas, Chikungunya virus into Italy or Usutu virus into Austria are examples of the vulnerability of our modern societies for arboviruses [3,8,9]. Sometimes the ways of introduction of arboviruses are obvious as in the case of Chikungunya virus in Italy, which was introduced by a viremic traveller returning from India. In other cases they remain obscure like the introduction of West Nile virus into the Americas [10]. Principally, two mechanisms of importation have to be discussed, the import by viremic vertebrates (humans, animals) and import by virus-bearing arthropods. While the introduction of new arthropod species, mainly mosquito species (e.g. *Aedes albopictus*, *Aedes japonicus*), is well-known and, in several countries, is under close observation, the risk and the importance of animal trade for the importation of arboviruses has not been studied extensively [11]. Vertebrate hosts, including humans, may play a role as vehicles for importation and the maintenance by amplifying various arboviruses.

Animals may be introduced into new areas intentionally or by their natural migration activities. The latter naturally varies tremendously depending on the annual migration patterns of the particular species. In Germany, for example, 1322 neozoon species have been registered since 1492, with 262 species that have established permanent and robust population numbers [12]. Regarding the establishment of a new arbovirus transmission cycle, these species may be suitable hosts to permit continuous viral transmission. Although not an arbovirus, the introduction of monkeypox virus into North America in 2003 via a Gambian giant rat from Africa is yet another example for animal trade contributing to the global spread of zoonotic diseases [13]. So far, the trade of animals has been rarely incriminated as means of importation of arboviruses. However, animals are traded for different reasons across the entire world, for food and food production, for scientific, educational and conservation reasons or as companion or, as in the case of the Gambian giant rats, pet animals, and also for touristic reasons [2,11,14]. The magnitude of global movement of animals is immense. From 2000 to 2004, more than a billion animals from 163 countries were legally imported into the United States of America [15]. This equals almost 600000 animals per day, but disease screening for arboviruses is mandatory only in limited cases. Likewise, hematophagous ectoparasites on imported animals which may act as vectors or which are already infected are likely to go unnoticed. Other data emphasise the potential of animal movement in the context of exotic pathogens. For the year 2002 it was estimated that 49 million amphibians and 1.9 million reptiles have been imported into the USA [16], providing a fair chance to import pathogens due to a lack of clinical symptoms in these animals [for review see [17]]. Introduction of animals by chance may play a major role in the introduction of arthropods. Several examples are prominent like the introduction of *Aedes albopictus *into the United States of America by used tyres or by bamboo plants into the Netherlands [8,18].

The last International Catalogue of Arboviruses listed more than 500 arboviruses and related viruses [[10]; http://www2.ncid.cdc.gov/arbocat/index.asp]. More than 150 of these are known to cause human and/or animal diseases. For many of those viruses, only limited information is available regarding their vector and host spectrum. Hence, we have chosen some prominent examples of important arboviruses causing human and animal diseases, which belong to the genera alphaviruses (family Togaviridae), flaviviruses (family Flaviviridae), and phleboviruses (family Bunyaviridae) to discuss the animal aspect in virus dispersal.

### Western Equine Encephalomyelitis virus

**Figure 1:**
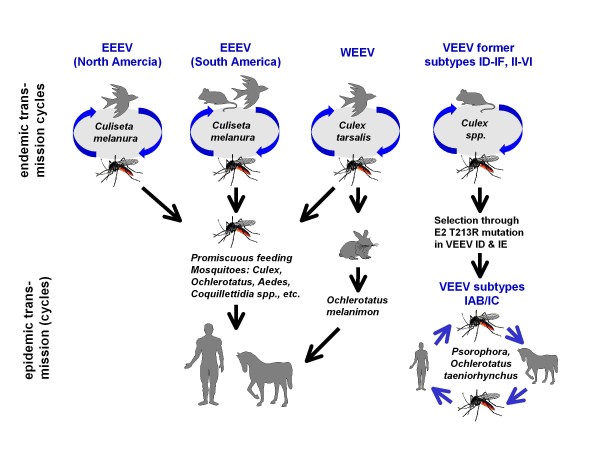
**Schematic drawing of the endemic and epidemic transmission cycles of eastern (EEEV), western (WEEV), and Venezuelan equine encephalitis viruses (VEEV)**.

Western Equine Encephalomyelitis (WEE) is caused by the Western equine encephalomyelitis virus (WEEV) which belongs to the genus Alphavirus in the family Togaviridae [19]. The virus occurs through most of the American continent, with virological and/or serological evidence of occurrence in the western parts of Canada, the U.S.A., in Mexico and throughout parts of Southern America (Guyana, Ecuador, Brazil, Uruguay and Argentina) [20,21]. WEEV is maintained in North America in a natural transmission cycle involving domestic and wild birds as the most important maintenance and amplifying vertebrate hosts and mosquito vectors, primarily *Culex tarsalis *[[21], Figure 1]. However, WEEV was isolated or detected in at least 14 mosquito species of the genus *Aedes *and six species of the genus *Culex *[22]. In South America, an additional mosquito-rodent cycle is postulated, involving mosquitoes of the genus *Aedes *and vertebrate hosts including rice rats (*Oryzomys spp*.), rabbits and introduced European hares (*Lepus europaeus*) [23-26]. Humans and horses do not develop viremias high enough to infect blood-sucking mosquitoes [19]. Therefore, they may not serve as maintenance or amplifying hosts and will not be able to sustain a transmission cycle in nature.

In humans, WEEV causes severe encephalitis with higher manifestation rates in children and in elderly persons. Fatality rates may be up to 5% [21]. WEEV is an important pathogen of horses where it causes a severe form of encephalomyelitis which may be fatal in up to 10 to 50% [21]. WEEV has constantly been declining in North America over the last decades and no veterinary nor human cases have been reported in 2009, with only one submitted mosquito pool testing positive for WEEV (http://diseasemaps.usgs.gov/; as of December 8^th ^2009). Less land irritation and consequently less breading opportunities for vector mosquito species have been claimed for the fading of the virus. To some extent the use of vaccines, which are available for equines but not for humans, might have attributed to this situation. Nevertheless, WEEV has been used to develop chimeric vaccines in combination with other alphaviruses such as Sindbis or eastern equine encephalitis viruses [[27]; see below].

WEEV may be introduced to Europe or to other parts outside the Americas by different routes. Infected adult mosquitoes or infected *Aedes *eggs (*Aedes dorsalis*) may be possible means of importation [22]. WEEV may also be introduced into Europe by viremic birds or by viremic rodents. As there are no major bird migration routes between the American and European continents, a natural introduction via infected birds seems unlikely. However, some long distance migrating bird species may share breeding grounds in the arctic with a slight chance of exchanging arboviruses, providing suitable vector mosquito species are present. Sick humans or horses do not develop viremias high enough to infect mosquitoes and thus cannot serve for the establishment of a new transmission cycle. Although studies on the vector competence of European mosquito species for the transmission of WEEV are missing, WEEV could be isolated from *Culex pipiens *and from *Aedes vexans*. Both mosquito species form a major part of the Central European mosquito fauna. For a natural transmission cycle, WEEV is dependent on passerine birds and possibly also on small wild mammals. Both groups of animals are abundant in Europe and although no data are available on the potential of European species to serve as natural maintenance or amplifying hosts, there are no obvious reasons to argue against a potential for transmission in European species. Hence, the risk of the introduction of WEEV into Europe seems to be low, although the required components for a natural transmission cycle of WEEV seem to be available (Table 1).

**Table 1:** Qualitative estimation of the impact of zoonotic arboviral diseases with a non-zero likelihood of evolving in response to animal trade, animal migration and climate change.

Arbovirus	Chances for dispersal	Major mode of dispersal	Chances for establishing new endemic foci (c)	Chances to be eliminated again (d)	Impact on public health (e)	Impact on veterinary public health (e)	Occurrence and distribution influenced by climate (f)
WEEV	Moderate	Long distance (viremic birds)	Moderate to high	Low to moderate	Low	Low	Yes
EEEV	Moderate	Long distance (viremic birds)	Moderate to high	Low to moderate	Low	Low	Yes
VEEV	Moderate to high (a)	Short distance (mosquitoes, rodents)	Moderate	Low to moderate	Low	Low to moderate (a)	Yes
WNV	Moderate to high	Long distance (viremic birds)	Moderate to high	Zero to low	Moderate to high	Low	Yes
JEV	Moderate	Long distance(viremic birds)	Moderate	Low to moderate	Moderate to high	Low to moderate	Yes
RVFV	Moderate to high	Short to long distance (livestock animals)	Moderate to high	Low to moderate	Moderate	Moderate to high	Yes
USUV	Moderate to high	Long distance (viremic birds)	Moderate to high	Zero to low	Negligible	Low	Yes
TBEV	Low to moderate	Short distance (ticks, rodents) (b)	Moderate to high	Zero to low	Low	Negligible	Yes
POWV	Low to moderate	Short distance (ticks, rodents) (b)	Moderate to high	Zero to low	Low	Negligible	Yes

(a) = Depending on the VEEV subtype involved.

(b) = When ticks are attached to birds, the respective viruses can as well be carried over long distances.

(c) = Because the mechanisms allowing a successful establishing of new endemic foci are poorly understood, the estimates provided are speculative despite for the viruses where this happened in recent history, e.g. WNV in America. Expansion of the geographic range of tick-borne TBEV and POWV mainly occurs on a different scale than with mosquito-borne arboviruses.

(d) = The general rule "the earlier the detection of the alien virus, the better the chance to successfully terminate it" applies for both mosquito- and tick-borne viruses, but due to the life cycle of mosquitoes and the availability of efficient larvicides and adulticides, their abundance can be better addressed with an integrated pest management and mosquito control program than fighting ticks in a tick habitat.

(e) = TBEV and JEV cause diseases in humans that can be prevented by applying safe and efficient vaccines. There is an inactivated vaccine available for the three equine encephalitis viruses and WNV.

(f) = The distribution of all arboviruses depends to a major part of the abundance of suitable vector species. Since their life cycle is strongly influenced by the weather, climate is an important issue in the occurrence and spread of arboviruses.

### Eastern Equine Encephalomyelitis Virus

Eastern equine encephalomyelitis (EEE) is caused by eastern equine encephalomyelitis virus (EEEV) which is also a member of the genus Alphavirus in the family Togaviridae. EEEV causes severe disease in humans, in horses and in some game animals [28]. In humans, fatality rates of up to 70% may be observed during some epidemics [29]. In horses, fatality rates of EEV infection may approach up to near 100% [19]. EEEV infections cause neurological disease in introduced bird species, like the sparrow, the ring-necked pheasant, the domestic pigeon and emus [30]. Emus and pheasants seem to serve as amplifying vertebrate hosts and epizootics in these animal stocks are observed with high fatality rates and enormous economic losses [31]. Besides birds, EEEV could be isolated from bats; however no transmission was detected in bats. Furthermore EEEV was isolated or infection was serologically proven in amphibians and reptiles. They can yield high viremias for several months and therefore are candidates for overwintering of EEEV virus in temperate climates [29,32]. An effective vaccine for use in equines is commercially available, but there is no approved EEEV vaccine for humans to date.

EEEV occurs in North and South America. While the natural transmission cycle(s) in South America are not well understood, transmission in Eastern North America is mainly dependent on ornithophilic mosquitoes of the species *Culiseta melanura *and passerine and wading birds of different species (Figure 1). The cycle is mainly maintained in coastal and inland swamps. Human and equine cases occur if large populations of mosquitoes of other species are abundant after heavy rains. These mosquito species may serve as bridging vectors, transmitting the EEEV obtained from viremic birds to horses and humans due to their more non-catholic feeding behaviour [[33], Figure 1]. EEEV was isolated from more than 20 different mosquito species, among them *Culex pipiens *and *Aedes vexans *which also occur in Central Europe and many other parts of the world (see: http://data.gbif.org/species/13452448/). The results of studies of transovarial transmission of EEEV in mosquitoes are conflicting. Probably EEEV is not transmitted via infection of eggs to the next mosquito generation while for *Coquilletidia perturbans *transovarial transmission could be proved [34].

The risk of an importation of EEEV into Europe or other areas outside of the American continent seems to be low. Basically, an importation seems possible via infected mosquitoes, infected birds (passerine, waders, farm birds like emus or pheasants) and also via infected reptiles and amphibians. As already mentioned for WEEV, no frequent migration of birds between the Americas and Europe exists. Therefore an introduction seems only possible as a result of human activities (e.g. trade, scientific, conservation, touristic activities). Although no studies on the vector competence of European mosquito species for EEEV are available, *Aedes vexans *and *Culex pipens *are among the most abundant mosquito species in Europe. However, at least in North America, *Culiseta melanura *seems to be the main vector for EEEV. The genus *Culiseta *is a rather species poor genus (five species worldwide), which has been claimed to be the reason for higher levels of genetic identity in viruses transmitted by *Culiseta *mosquitoes than in viruses that mainly use *Culex *or *Aedes *vector species [35]. In contrast to WEEV, where no clinical symptoms in birds seem to occur, EEEV seems to cause neurologic disease and haemorrhagic disease with death in many species of non-American wild birds. Therefore, the introduction and establishment of EEEV in the European bird populations would probably cause high death rates in birds and would likely be detected at an early time-point after introduction (Table 1). As for WEEV, the basic factors for the establishment of a natural cycle seem to be available in Europe also for EEEV.

### Venezuelan Equine Encephalomyelitis Virus

Venezuelan equine encephalomyelitis is caused by a complex of viruses (Venezuelan equine encephalomyelitis virus, VEEV) which belongs to the genus Alphavirus in the family Togaviridae. The complex includes seven different virus species and a number of subtypes and varieties [36]. VEEV occurs mainly in tropical and subtropical regions of the Americas and circulate endemically between mosquitoes of the genus *Culex *(*Melanoconion*) and rodents (*Oryzomys*, *Proechimys*, *Sigmodon*, *Peromyscus*, *Heteromys*, *Zygodontomys*) [[37], Figure 1]. However, some species of birds, mainly herons, also develop high and prolonged viremias and thus can infect blood-sucking mosquitoes. Therefore these birds may serve as maintenance and amplifying hosts on particular occasions [37]. Other wild or farm animals do not seem to replicate VEEV in virus titres high enough to serve as hosts for maintenance of transmission cycles. Also humans infected with epidemic VEEV strains develop high titres and may therefore play a role as maintenance and amplifying hosts [38,39].

Major VEE epidemics occur sporadically or periodically when epidemic strains of the subtypes IAB and IC spill over into competent mosquitoes of the genus *Aedes *and *Psorophora *which have a peridomestic/peri-agricultural behaviour and may transmit VEEV to equines, donkeys and mules. Equids develop high virus titres and therefore may serve as amplifying hosts for VEEV. An equine-mosquito-cycle may induce an extensive virus circulation with a spill-over to humans and cause epidemic VEE (Figure 1). Epidemic VEE in humans is a highly incapacitating dengue-like illness which in a small part of infected people, mainly in children, may cause severe encephalitis with fatality rates of 1 to 3% [40]. There is no specific treatment available to cure the disease and no human vaccine to prevent it. A vaccine for equids, however, can be purchased.

The epidemic occurrence of VEEV during the last two decades shows that it is highly variable in nature and that single amino acid changes in the viral genome may cause major changes in vector competence of mosquitoes or in the pathogenicity in equids [41-43]. Studies also show that epidemic strains of VEEV adapt to one of the important epidemic bridge-vectors (*Ochlerotatus taeniorhynchus *formerly *Aedes taeniorhynchus*) and replicate to higher titres in this mosquito species than in mosquitoes involved in endemic transmission (*Melanoconion*) [44]. The introduction and establishment of VEEV into Europe may be possible via infected mosquitoes, rodents, birds (herons), horses and humans (Table 1). The establishment of enzootic viruses needs susceptible rodents and transmitting competent mosquitoes. While in Central and Southern America, mainly rodents of the subfamily Sigmodontinae are involved as maintenance hosts, data on the replication of different VEEV subtypes in European rodents of the subfamilies Murinae and Arvicolinae are not available. Whether mosquitoes of the genera *Culex *and *Aedes *in Europe are competent for VEEV has not been studied so far. However, American strains of *Aedes albopictus *were found to be capable of transmitting VEEV [45,46]. Therefore, at least a limited peri-domestic or urban (human-mosquito-human) transmission cycle with epidemic VEEV strains seems possible (Table 1). However, for larger epizootics and epidemics of VEEV, larger populations of non-immune equids are a pre-requisite for the initiation of the epidemic transmission cycles.

### West Nile Virus

West Nile virus (WNV) is a member of the Japanese encephalitis group of the genus Flavivirus in the family Flaviviridae. The evolutionary origin of WNV seems to be in Central Africa, from where it spread over various parts of the world and locally new genotypes emerged [47]. Actually five genetic lineages are recognized, from which only lineage 1a is distributed worldwide while the other lineages and sub-lineages exhibit a more local geographic distribution [48]. WNV causes a febrile illness or encephalitis in humans and horses [49]. In humans the fatality rate of WNV CNS infections ranges from 5 to 10% with higher rates in elderly people or those with additional underlying diseases [50]. The introduction of WNV in the Americas caused a high fatality rate in different American species of birds (e.g. Corvidae), while fatalities by WNV infections in wild birds in the Old World have not been reported so far [51]. However in Israel epizootics in geese were repeatedly reported during the last decades.

**Figure 2:**
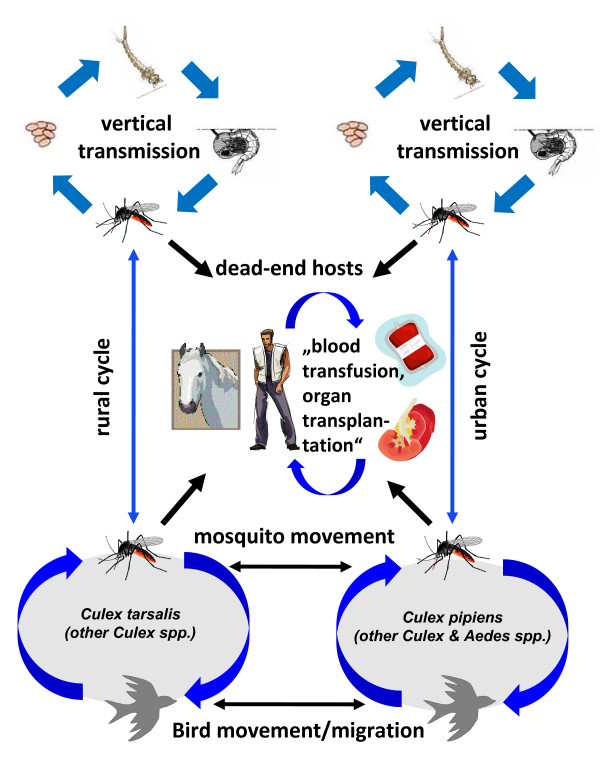
**Schematic drawing of the transmission cycles and possible modes of dispersal of West Nile virus**.

Like other members of the Japanese encephalitis serogroup, WNV in nature is maintained in a bird-mosquito cycle (Figure 2). WNV was isolated or detected in at least 43 species of old world mosquito species, mainly belonging to the genus *Culex *[52]. The importance of other mosquito genera and species (*Aedes*, *Anopheles*, *Aedomyia*, *Mansonia*, *Coquilletidia*) and of hard and soft ticks (*Hyalomma*, *Dermacentor*, *Rhipicephalus*, *Amblyomma*, *Argas*, *Ornithodoros*) for the endemic and epidemic transmission cycles remains to be determined [53]. Various birds, mainly passerines serve as primary vertebrate hosts of WNV [54,55]. WNV infections were also detected in rodents and other small mammals, however, these animals do not seem to produce viremias high enough for maintaining the transmission cycle. Moderate viremias, however, were detected in horses and in lemurs in Madagascar [55]. These animals may support the virus transmission cycle under local ecological conditions. In one study a frog (*Rana ridibunda*) was found to be viremic and was able to transmit the virus to blood-sucking *Culex pipiens *[56]. Therefore, also a frog-mosquito-frog-cycle seems to be possible under certain ecological conditions.

WNV is an often cited example of a dispersing arbovirus since it invaded into North America in 1999 [10]. From the original point of invasion (New York) the WNV dispersed within a few years over the total continental U.S.A. and Southern parts of Canada and also migrated into Central America and parts of South America. The main way of migration is thought to be via migration of birds. Several bird species (house sparrow, blue jays, American robins) may have played an important role in the distribution of WNV in the Americas. Additionally, there is evidence that different mosquito species were important in different parts of Northern America for the transmission of WNV, and that a more efficiently replicating strain evolved in 2003 entirely replacing the originally introduced WNV strain in North America [57].

The exact way of introduction of WNV into North America is still unclear. Several additional factors are discussed which improved the establishment and transmission of WNV in this new environment (Table 1). Among them are the introduction and geographic dispersion of large and WNV non-immune populations of the house sparrow, which served as a very efficient maintenance host for WNV, the availability of a very competent vector (*Culex pipiens*), climate warming, and perhaps also the decline of infections with the closely related St. Louis encephalitis virus, an indigenous virus of the Japanese encephalitis serogroup in the Americas [9]. However in Europe, instead of all discussions on the geographic dispersion and introduction into new regions, no clear increase of the range of distribution of WNV can be observed. Since the early 1970s, when the virus was detected in Czechoslovakia, no extension of distribution further northward was detected despite many efforts to detect WNV in Central and Northern Europe after the introduction in the Americas, although competent vectors as well as maintenance and amplifying hosts for WNV seem to exist in Central Europe and repeated introductions into Central Europe have occurred [58,59]. In a risk assessment of the introduction of WNV into the Galapagos Islands, four modes of introduction are discussed: introduction via infected humans, via infected migratory birds, via infected mosquitoes, and via human-transported host vertebrates [60]. The introduction via infected humans could be excluded, as humans do not develop viremias high enough for infecting mosquitoes. The analysis showed that the highest risk of an introduction of WNV is infected mosquitoes un-intentionally transported in airoplanes carrying tourists. Also the introduction of WNV via infected eggs or larvae in tyres seemed to be of importance. Instead, the introduction of WNV via migratory birds or via infected chickens seemed to be at least one magnitude lower than due to airoplane-transported mosquitoes. In the case of optimized conditions the introduction of WNV may most probably happen due to migratory birds or via carrying of infected mosquitoes from endemic areas via human transport activities. Therefore, the migratory bird routes and the main transport routes from endemic southern and South-eastern Europe may be most important for continuous surveillance [48,61,62].

### Japanese Encephalitis Virus

**Figure 3:**
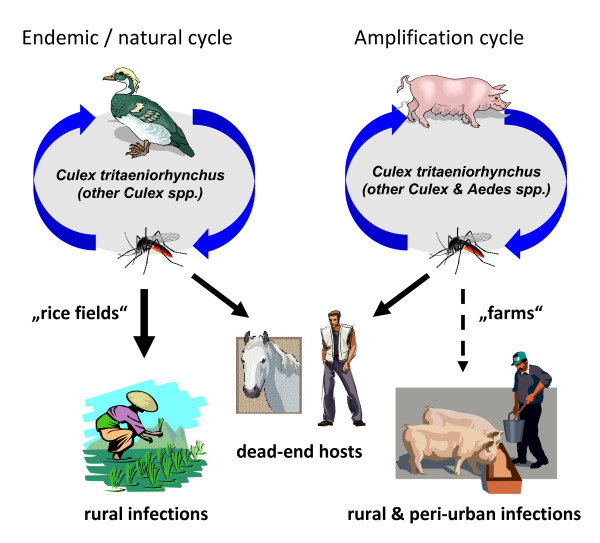
**Schematic drawing of transmission cycles and rural as well as peri-urban infections of animals and humans with Japanese encephalitis virus**.

Japanese encephalitis virus (JEV) is a member of the similarly named serogroup in the genus *Flavivirus *of the family Flaviviridae. JEV is transmitted in a natural transmission cycle involving mosquitoes of the genus *Culex *and water birds (mainly egrets and herons) [63]. Actually five lineages of JEV can be distinguished which is of importance for epidemiological studies [9]. Currently, JEV is the most important mosquito-transmitted arbovirus, causing encephalitis in the world. An estimated 30,000 to 50,000 human cases occur every year [64]. Up to 30% of all ill humans die, and about half of the surviving patients show persisting, life-long neurologic sequelae [65]. JEV infects a number of different animals, among them dogs, ducks, chicken, cattle, bats, snakes and frogs. Humans and horses may develop a severe and fatal form of encephalitis. However, the viremia titres in humans and horses are not high enough to serve as transmission hosts (Figure 3). In contrast, pigs develop high viremias and they therefore serve as amplification hosts for bridge vectors to initiate epizootics and epidemics [66].

The natural transmission cycle mainly involves mosquitoes of the genus *Culex*. The primary vector is *Culex tritaeniorhynchus*, which is associated with rice paddies and irrigated crop fields in whole Southeast Asia. *Culex tritaeniorhynchus *feeds on water birds and on larger mammals, also on pigs and therefore transmits JEV to this important amplifying host, and also to equids and to humans. Other *Culex *species, like *Culex pipens*, *Culex vishnui *and *Culex bitaeniorhynchus *may play a local role for the transmission of JEV (Figure 3). The natural vertebrate hosts of JEV are ardeid birds, mainly the black-crowned night heron (*Nycticorax nycticorax*) and the Asian cattle egret (*Bubulcus ibis coromandus*) [67]. There is evidence that JEV is also transmitted transovarially in *Culex tritaeniorhynchus*. Therefore, an enzootic or an epizootic cycle may be initiated from mosquitoes directly after diapause. The invasion of JEV in new areas in Southeast Asia during the last decades has been mainly associated with the increase of human populations and, consequently, in increasing areas of rice paddies and pig farming [68]. JEV recently expanded also in higher altitudes in the Kathmandu valley of Nepal and into New Guinea and to the Torres Straight and to Northern Australia [69,70].

Japanese encephalitis virus shows a clear tendency of expansion. One mechanism of spread involves the air transport of infected mosquitoes. This method of spread was shown by the introduction of JEV into Pacific islands like Guam or Saipan [71,72]. A recent study showed that the potential risk of an introduction of JEV into the west coast of the United States is possible. Competent vectors and pigs as amplifying vertebrate hosts are available in moderate numbers. However pigs in California do not live in residential environments as in Asia, but in large pig farms, which are dispersed throughout the state. Therefore, the risk of a spread of introduced JEV may be lower as in the agricultural areas of Asian countries. However, the feral pig production farms provide sufficient non-immune populations for an amplification and potential spread of JEV in California. As the viremia in pigs may be prolonged, also the transport of pigs to new locations/farms may provide a way of transport for the spread of JEV for small and moderate distances. Also, a further introduction into central Asia and even into eastern and Central Europe seems possible (Table 1). Birds may also play a critical role of transporting over long distances and pigs may be responsible for the local distribution of the virus. JEV is one of the arboviruses with a high potential of expansion into virgin areas [73].

### Rift Valley Fever Virus

Rift Valley fever (RVF) is a disease which was first described as an entity during an epizootic outbreak in 1930 - 1931 in Kenya [74]. There, the etiologic agent, Rift Valley fever virus (RVFV) causes severe disease, stillbirth and often death of cattle, sheep and goats [75]. Only in the 1950s, first cases of an undifferentiated fever in humans were associated with infection of RVFV. Apart from the original outbreak, the pathogenic potential of RVFV for humans was described in detail during outbreaks in the 1950s [74]. In 1975, during a large outbreak of RVF in South-Africa, the first fatal human cases were described and the virus was reclassified as a hemorrhagic fever virus [76]. Until 1977, RVFV outbreaks were limited to Sub-Saharan Africa. In 1977 an epizootic RVF epidemic occurred in Egypt, for the first time north of the Saharan desert. During this epidemic more than 200,000 human cases with 600 fatalities were registered. Besides hemorrhagic manifestations the virus caused retinitis with blindness, hepatitis and encephalitis [77,78]. During the late 1980s a new extension of the geographic range of RVFV into western Africa was detected. And again in 2000, RVFV caused an epizootic and epidemic in Saudi-Arabia and Yemen, the first time that RVF was detected outside of Africa [79,80].

**Figure 4:**
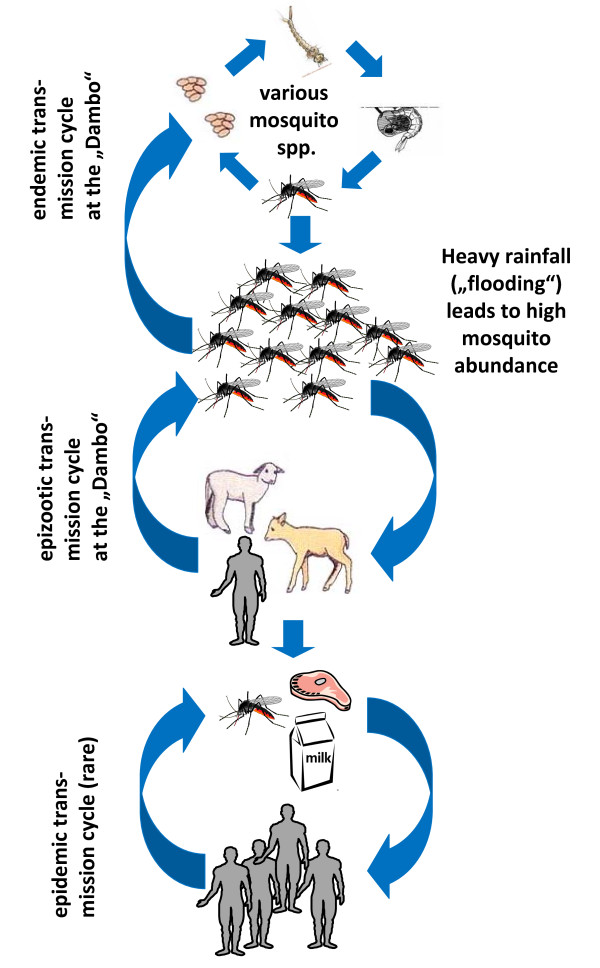
**Schematic drawing of the development from an endemic transmission cycle through an epizootic transmission cycle to epidemic transmission of Rift Valley fever virus**.

RVFV belongs to the genus *Phlebovirus *of the family Bunyaviridae. It is transmitted in an enzootic cycle among wildlife and mosquitoes [81]. RVF is a promiscuous virus, using a number of different mosquito species belonging to different genera (*Aedes*, *Ochlerotatus*, *Stegomyia*, *Anopheles*, *Culex*, *Neomelaniconion*, *Eretmapodites *and others) as vectors [[74], Figure 4]. The role of most of these mosquito species for the maintenance of the enzootic cycle is unclear. Probably the most important way of maintaining the enzootic cycle is the transovarial transmission in mosquitoes, mainly of the genus *Aedes*. *Aedes macintoshi *seems to play a major role in Eastern Africa [82]. *Aedes macintoshi *lays infected eggs into the ground and these eggs need one or more severe flooding to hatch. Therefore an inter-epidemic period (low mosquito population, low number of cases of RVF) and an epidemic period (high populations of mosquitoes and high numbers of sick animals and of human cases) can be distinguished. The occurrence of epidemic periods is clearly associated with heavy rains which are closely linked to warming of the Indian Ocean during the El Nino Southern Oscillations (Figure 4). The impact of climate change on Rift Valley fever virus infections is clearly relevant and has been subject to a recent review [83]. Wild and domestic animals are infected and serve as amplification hosts to create more infected mosquitoes. RVFV may be transmitted to other mosquito species which serve as bridging vectors to other wild and domestic animals and to humans which may cause further amplification of the transmission cycle [[84], Figure 4].

These examples show that RVFV, without any doubt, is one of the most aggressive migrating arbovirus. The routes of dispersal detected so far seem to be in parallel with the great migration routes of camels. Therefore, there is some good evidence that viremic, but non-symptomatic infected camels transported the virus to Egypt and possibly also to the Arabian Peninsula [85]. As also humans may serve as amplifying hosts, the introduction of RVFV by viremic humans seems possible and probable. In 2008, one case of RVF was diagnosed retrospectively in Germany in an ill woman, who had returned from Africa [86]. However, few data exist on the vector competence of European mosquito species for RVFV. Initial results on the dissemination rates in some infected mosquito species tested, suggest that most of these may serve as vectors [87]. Likewise, an introduction into the United States may be possible, as was seen for West Nile virus in 1999. Several ways of introduction were discussed, and the risk of importation into the US by infected animals, by infected people, by mechanical transport of infected insects, intercontinental wind-borne transport of RVFV- bearing insects, and also by intentional introduction and release of RVFV were assessed [88]. Studies on the vector competence of Northern American mosquitoes showed that several common species (*Aedes vexans*, *Culex erraticus*, *Culex nigripalpus*, *Culex quinquefasciatus*, *Culex salinarius*) can be infected and develop systemic infection. However, only *Aedes vexans *and *Culex erraticus *developed virus titres which were high enough to transmit the virus to laboratory animals [89,90]. Therefore with the presence of competent vectors and large populations of naive, non-immune wild and domestic ruminants (and possibly humans), the necessary factors exist in North America to establish a transmission cycle (Table 1). Similar studies for Europe are still missing. However, there is little doubt that vectors and ruminants are present in Europe to allow establishing of at least temporary enzootic transmission cycles (Table 1).

### Usutu virus

Usutu virus (USUV) belongs to the Japanese encephalitis serogroup within the mosquito-borne cluster of the genus *Flavivirus *in the family Flaviviridae [91]. It was originally isolated from mosquitoes of the genus *Culex *in South Africa in 1959. Since that time the virus was isolated several times from mosquitoes, rodents and birds throughout Sub-Saharan Africa [92]. There has been some limited information that USUV may be the etiologic agent of a mild human disease with fever and rash [93]. In 2001, USUV suddenly emerged in the area of the Austrian capital Vienna and caused widespread deaths among the population of Eurasian blackbirds (*Turdus merula*) and some other bird species. USUV could be detected the following years and its area of distribution extended into south-east (Hungary), south (Italy), west (Switzerland), and north (Czech Republic, Poland) of the original location of emergence where it also caused mortality in birds [55,94,95]. In 2009, USUV was shown to exhibit human pathogenicity when it was for the first time detected to cause neuroinvasive infection in two patients with immune deficiency (orthotopic liver transplantation, B cell lymphoma) in Italy [96,97]. USUV, most probably was introduced into Austria via viremic birds returning from their winter migration from Africa to Europe. Another possible way of introduction could be the transport of virus-infected mosquitoes from Africa to Austria via airoplane, as the location of emergence in Austria, Vienna, harbours the largest international airport in Austria.

USUV is thought to be maintained in nature in a mosquito-bird transmission cycle. In Africa ornithophilic mosquitoes of the genera *Culex*, *Coquillettidia *and *Mansonia *are thought to be the main vectors. In Austria, *Culex *spp. may play a major role, although USUV so far has not been isolated from mosquitoes but has been detected in overwintering *Culex pipiens *pools by real time RT-PCR (our own unpublished results). There seems to be a mode of adaptation of the virus to the new bird species and/or to the new mosquito species in Europe. After high mortality rates in blackbirds during the first two years of emergence of USUV, in the following years increasing rates of seropositive birds were detected in Austria which gave evidence for a continuing circulation of USUV with a somewhat lower pathogenicity, inducing an herd immunity in the bird populations [94].

USUV appears as an impressive example for the introduction and permanent establishment of a so-called "tropical" arbovirus in moderate climates. In a recent study, it was argued that USUV is mainly maintained in a natural cycle in areas of Austria with a minimum of at least ten hot days (> 30°C) [98]. In this simulation it is predicted that USUV will become endemic in larger parts of Central Europe until the end of the century. According to the presented model, optimal environmental conditions for outbreaks of USUV will occur in about 10 years from now on [98]. Whether USUV will develop in a similar way as WNV did in the Americas remains to be seen in the future. And even more striking is the question whether the closely related WNV would behave in a similar way.

### Tick-borne encephalitis virus

**Figure 5:**
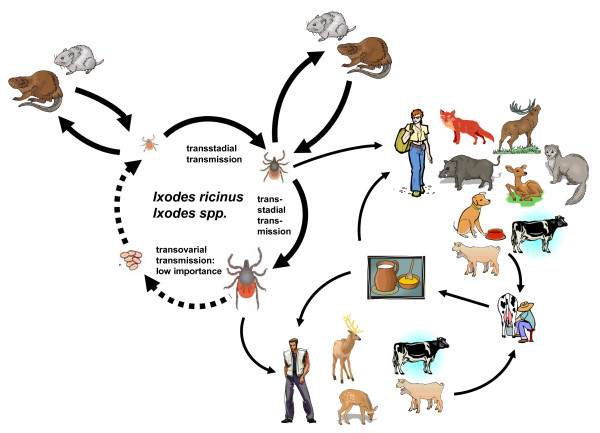
**Schematic drawing of the transmission cycle of tick-borne encephalitis virus**.

So far, only the invasive potential of mosquito-borne arboviruses has been discussed. The example of tick-borne encephalitis virus (TBEV) shows that also tick-transmitted arboviruses may be able to invade new areas. TBEV is a flavivirus of the tick-borne group of the genus *Flavivirus *in the family Flaviviridae [99]. It is distributed in the northern hemisphere of Europe and Asia. There, it is transmitted in nature by hard ticks (Ixodidae, almost exclusively *Ixodes ricinus *and *Ixodes persulcatus*). The natural vertebrate hosts of TBE virus are small rodents of the genera *Myodes *and *Apodemus*, although other Rodentia or Eulipotyphla (formerly: Insectivora) may contribute to the natural transmission cycle [[99]; see Figure 5]. In contrast to mosquitoes, ticks do not depend on a sufficient viremia of the infected host to take up an arbovirus. While blood-feeding until repletion of a mosquito is a question of a few minutes, ticks are attached to their host for up to a week. So-called saliva-assisted transmission (SAT) is the indirect promotion of arbovirus transmission via the actions of tick saliva molecules on the vertebrate host [100]. The skin site where ticks feed is highly modified by the pharmacologically active molecules secreted in the tick saliva. This phenomenon is crucial in maintaining a threshold level of infected tick individuals in a tick population through a mechanism known as co-feeding. Co-feeding is facilitated through feeding of a number of ticks in close proximity on the host skin and mediated via the tick saliva. During co-feeding, pathogens such as TBEV are transferred from one tick to another [101]. Adults and immature ticks (either larvae or nymphs) feed on the same reservoir host, mostly rodents, thus transmitting and maintaining the arbovirus between the different life stages of the vector. Co-feeding and thus the TBEV prevalence in an enzootic focus depends on the simultaneous presence of nymphs and larvae (and adults) on the vertebrate host. As for *Ixodes ricinus *in Europe, larvae become active above 10°C while nymphs start searching for suitable hosts at 7°C. This means, among many other factors, that a fast warming in spring will be beneficial for co-feeding and in turn will result in higher numbers of TBEV-positive ticks [102,103].

TBEV is the most important tick-transmitted arbovirus of human pathogenicity in Europe and Asia [104,105]. An estimated 10000 to 15000 human cases occur annually with a fatality rate of 1% (Western subtype) to up to 20% (Far Eastern subtype) [106,107]. The geographic origin of the emergence of TBEV has been known due to comparative sequence studies for several years. These studies show that TBEV originated somewhere in the Siberian or Far Eastern area [108]. From there, the virus dispersed to the south and to the west. During its movement new subtypes and viruses evolved: the western subtype of TBEV and louping ill virus on the British islands, in Spain and in Norway [109]. The movement into the eastern direction finally ended in the evolution of the Far Eastern subtype (in China and Japan) and Powassan virus which is prevalent in Russia and in Northern America [109,110]. More additional available viral sequences showed that TBEV was introduced at least three different times to Japan alone during the last several hundred years [110]. However, not much is known about the possible ways how TBEV disperses over long distances. As humans and domestic animals (cattle, goat, and sheep) and game animals (deer, boar, fox, and wolf) do not develop high viremias they are unable to re-infect ticks during blood-sucking (dead-end hosts). Therefore, viremic humans and animals seem not to play a role in transporting TBEV into new areas. Mainly goats and, to a lesser extent, also cattle and sheep may transmit the virus via milk to their offspring. In case of trading raw milk and cheese products, the virus can be transported and can infect humans [111], but the mode of dispersal cannot result in establishing a new TBEV focus (Table 1). Scandinavian researchers showed that the migration of birds could play a major role for the migration of tick-borne viruses. They found ticks (mainly larvae and nymphs of *Ixodes ricinus*) on every 30^th ^bird which migrated in autumn from Northern Europe towards the South. About one out of 2200 migrating birds carried a TBEV-infected tick [112]. These data offer new insights into the potential migration of TBEV over long distances. However, no phylogenetic relationship between TBEV strains from northern Europe and from Central Europe could be detected. A new phylogenetic study of more than 160 TBE virus strains from the Siberian region shows that TBEV in Russia moved along the main transport routes in Russia [113]. At least two introductions from Siberia into western direction are detectable. These invasions of TBEV into western parts of Russia and the Baltic countries can be associated with major human activities, the construction of the first land road into Siberia and the construction of the Trans-Siberian Way [113]. The anthropogenic factor, i.e. human activity therefore seems to be the most important factor for the distribution of TBEV into the western parts of Europe. Potential ways of transport may be viremic rodents which follow humans on the main routes or virus-infected ticks which are carried by humans or human-associated animals (Table 1).

### Powassan virus

Powassan virus (POWV) is the sole member of the tick-borne encephalitis serological complex of flaviviruses in North America. It received its name after the town Powassan in Ontario, Canada, were it was isolated from the brain of a child deceased after encephalitis in the late 1950^th ^[114] and a couple of years earlier from ticks collected in Colorado, USA [115]. The latter was initially name deer tick virus and listed as a distince virus species DTV, but recent molecular analyses placed DTV as a genotype of POWV [116-118]. More interesting is the ecology of POWV, since it seems to exist in three rather discrete enzootic cycles: *Ixodes cookie *and woodchucks and mustelids; *Ixodes marxi *and squirrels; *Ixodes scapularis *and white-footed mice [119]. POWV has also been found in considerable numbers in *Dermacentor *ticks, namely *D. andersoni *and *D. variablis *but the corresponding enzootic cycle has not ben explored in further detail. Vertical transmission of POWV was observed in *Ixodes scapularis *[120]. The current distribution of POWV with parts of Canada and the USA, as well as Parts of Russia is interesting because it suggests that the Bering Strait had to be crossed at least once in history to explain the current geographical range of POWV. Phylogenetic studies of the TBE serogroup viruses place an Eurasian progenitor as common ancestor for POWV in North America [121]. One way of how POWV could have been introduced is by animals moving across the Bering land bridge during a recent glacial period or by migrating birds (as discussed above for TBEV). The tight clustering of Russian and Canadian strains suggests a rather recent introduction perhaps along with American mink that were imported to support fur trade [122]. So this is likely another example of the emergence of an arbovirus by animal trade.

It is interesting that for other tick-borne arboviruses, similar results on the importance of human activities for the spread into new, non-endemic areas are evolving. Kyasanur Forest virus, a virus related to TBEV is limited to some regions (Karnataka) in India [123]. However, a few years ago a closely related tick-borne virus, Alkhurma virus, was detected in cases of hemorrhagic fever in Saudi Arabia [123]. Also for this virus, mainly human activities are suspected for the recent dispersion by viremic animals or virus-infected ticks from India to the Arabian Peninsula. For another tick-borne arbovirus, Crimean-Congo Hemorrhagic Fever virus, human activities and changes in agricultural practices seem to be a major factor for emergence and distribution during the last years [124]. Louping ill virus is a relative of TBEV. This virus probably evolved on the British Isles from the introduced TBEV strain(s) [125]. Louping ill virus was transported with human activities to the Iberian Peninsula where a new subtype of the virus has evolved since then (Spanish sheep encephalitis virus). It was also transported to Norway where it is now dispersing, possibly due to climatic changes, to the north [125].

## Conclusions

Arboviruses are maintained in nature in complex transmission cycles between arthropods and vertebrates. They have developed strategies of adaptation and evolution to spread into new areas and eventually become established. Several recent examples show, that tropical arboviruses are capable to spread to countries with moderate climates. While bird-associated mosquito-borne viruses seem to be transported mainly by migrating birds, human activities (travel, trade) play a major role for arboviruses where humans play a role as natural vertebrate hosts. Also for tick-borne arboviruses, mainly human activities seem to contribute to the spread over long distances and the establishment in new ecosystems changed by human activities. In most cases of newly emerging zoonotic arboviruses, the ways of introduction remain obscure. Future research should aim at exploring the circumstances of these events. A better understanding of how arboviruses travel and why they become established in other geographic areas will be of great benefit for human and veterinary public health, because it may help to prevent devastating outbreaks of arboviral diseases in humans and animals.

## Competing interests

The authors declare that they have no competing interests.

## Authors' contributions

Both authors contributed equally to this work.
